# A presumptive case of cutaneous emergomycosis in a female patient with HIV – Maseru, Lesotho

**DOI:** 10.4102/sajid.v37i1.415

**Published:** 2022-10-26

**Authors:** Waheeba M.H. Madani, Wayne Grayson

**Affiliations:** 1 Department of Internal Medicine, Queen Mamohato Memorial Hospital, Maseru, Lesotho; 2 Department of Internal Medicine, Faculty of Health Science, University of KwaZulu-Natal, Durban, South Africa; 3 Department of Internal Medicine, Ministry of Health, Mafeteng, South Africa; 4 Department of Pathology, Faculty of Health Sciences, University of the Witwatersrand, Johannesburg, South Africa; 5 Department of Histopathology, Ampath Laboratories, Johannesburg, South Africa

**Keywords:** emergomyces africanus, emergomycosis, Emmonsia, fungal infection, HIV, skin

## Abstract

Emergomycosis is a recently described emerging opportunistic fungal infection among individuals living with HIV, in whom it is a cause of significant mortality and morbidity. This article retrospectively reports on a presumptive case of extensive cutaneous emergomycosis in a young immunocompromised patient from Lesotho. The histopathological features on skin biopsy were in keeping with emergomyces infection. The lesions responded to treatment with amphotericin B and oral fluconazole.

**Contribution:**

This case contributes to the existing evidence that as an emergent opportunistic infection, emergomycosis is possibly widespread in Africa but the true extend of the disease is not fully defined. This is further aggravated by the diagnostic difficulty as a result of limited resources in some areas in the region.

## Introduction

A number of opportunistic infections caused by invasive fungal pathogens have emerged over the course of the HIV pandemic, resulting in significant mortality and morbidity.^1^ These AIDS-defining infections include cryptococcosis, histoplasmosis and talaromycosis (formerly penicilliosis). The recent recognition of *Emergomyces africanus* as an important HIV- and AIDS-associated fungal pathogen has expanded this disease spectrum. The journey to its proper identification is a particularly interesting one. In 1994, cultures from an immunocompromised woman in Italy yielded a *Histoplasma*-like fungus closely related to *Emmonsia crescens* and *Emmonsia parva*. Later genetic analysis in 1998 resulted in this fungus being named *Emergomyces pasteurianus*.^2^ The systemic disease caused by these three species under the *Emmonsia* genus was known as disseminated Emmonsiosis.^3^ In 2013, a thermally dimorphic fungus thought to share similarities with *Emergomyces pasteurianus* was isolated in South African laboratories using molecular identification methods. It was found to be the causative agent in most of the systemic dimorphic mycosis in individuals living with HIV in South Africa.^4^ In recent years, DNA-based genetic analysis resulted in a new classification of the family Ajellomycetaceae. Consequently, *Emmonsia parva* became part of the genus *Blastomyces parvus, Emmonsia sola* and *Emmonsia crescens* formed the *Emmonsia* genus, and a new genus, *Emergomyces*, then emerged. The latter includes all *Emmonsia*-like dimorphic fungi related to *Emergomyces pasteurianus*.^5,6^ To date, five species within the genus *Emergomyces* have been identified in different parts of the world, namely *Es. pasteurianus* reported in Spain, South Africa, France, Italy, China and India; *Es. europaeus* as a single case from Germany; *Es. canadensis* in the United States and Canada; *Es. orientalis* in China; and *Es. africanus* in Southern Africa.^2^ In a recent update in nomenclature, *Emmonsia sola* and *Emmonsia crescens* were reclassified into the closely related *Emergomyces* genus.^7^

Prior to 2013, when cases of emergomycosis were first reported from South Africa, the disease was not known to be endemic to sub-Saharan Africa.^8^ When infection with the genus *Emergomyces* (formerly *Emmonsia*) was identified as a cause of disseminated disease in patients in Western Cape province of South Africa in 2013, it was initially thought to follow a geographical distribution.^9^ Additional cases, however, were subsequently diagnosed in six of South Africa’s nine provinces.^10,11^

Up until 2019, when a case was reported from Uganda; South Africa was the only country in Africa in which emergomycosis was known to have occurred. This article reports a presumptive case of disseminated cutaneous emergomycosis in a patient living with HIV from Lesotho, who was treated successfully with amphotericin B and oral fluconazole.

## Case report

In August 2018, a17-year-old female patient living with HIV presented to the Queen Mamohato Memorial Hospital (QMMH) in the Lesotho with a 10-month history of progressive weight loss and skin lesions, mainly involving the face and oral cavity. She had attended local hospitals and clinics with the same skin lesions multiple times in the past. The lesions commenced as gingivostomatitis and facial patches, which then progressed to involve the entire body. Clinical differential diagnoses entertained included dermatitis, viral exanthem, a bacterial skin rash, syphilis and Kaposi sarcoma. She had received multiple courses of topical and systemic antibiotics, antiviral therapy and corticosteroids but there was no satisfactory improvement. She had been on antiretroviral therapy (ART) (tenofavir, lamivudine and efavirenz) since 2014 and she was in good health until her illness started. The latter was heralded by significant weight loss, fever and several admissions to a local hospital with pneumonia. Over this period, monitoring of her HIV infection became erratic, as she was unable to attend her ART clinic.

**FIGURE 1:**
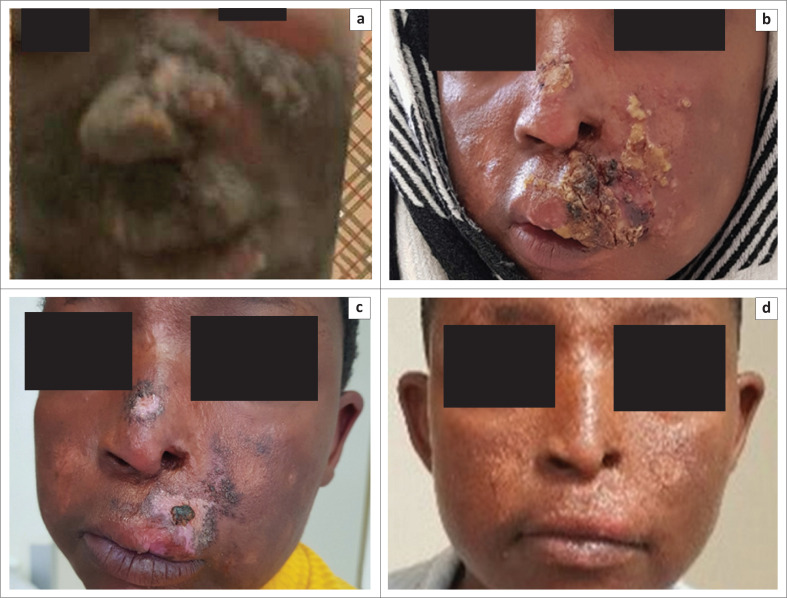
Clinical images of presumed case of emergomycosis, with (a) large crusted facial lesions at initial presentation; (b) a marked improvement following 3 months of oral treatment; (c) minimal residual disease some 7 months later; and (d) complete disappearance of the cutaneous lesions at one year, with mild residual scarring present.

At presentation to the dermatology clinic at the QMMH, she complained of odynophagia and the disfiguring skin lesions. Physical examination revealed a body mass index (BMI) of 18.1, an inability to open her mouth and crusted blackish and yellowish nodules and plaques involving the face (Figure 1a), limbs and trunk, with stigmata of secondary infection. A skin biopsy was performed, and the patient was admitted to hospital for systemic antifungal treatment after the biopsy results became available. Upon admission, baseline blood results showed mild hypokalaemia, normal renal function, elevated liver enzymes and a normal chest radiograph. The viral load (VL) was of 98 970 copies/mL.

**FIGURE 2:**
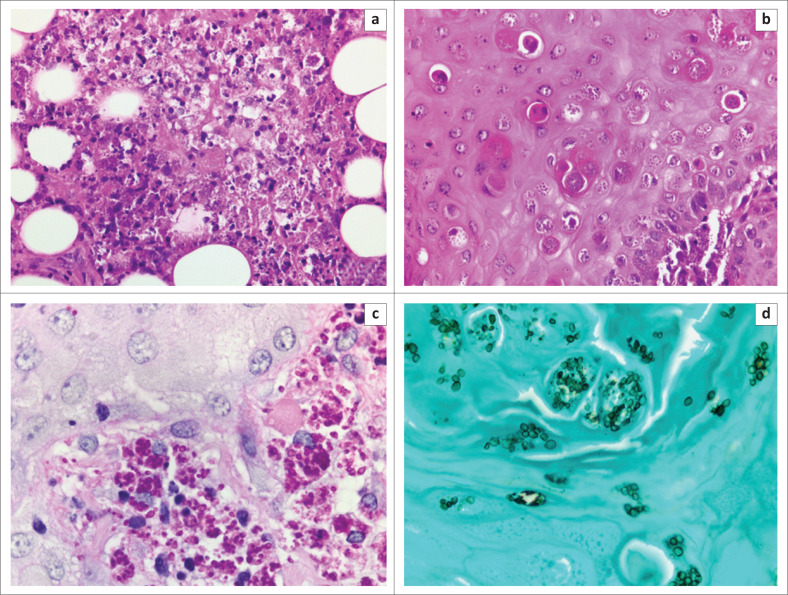
Histopathology of presumed *Emergomyces africanus* infection, with (a) deep dermal and superficial subcutaneous histiocytic and neutrophilic infiltrate in response to the fungal yeasts, with background karyorrhectic debris (haematoxylin and eosin, × 400); (b) pseudoepithelomatous hyperplasis and attempted transepidermal elimination of intracellular and extracellular yeasts (haematoxylin and eosin, × 400); (c) periodic acid-Schiff stain highlighting the predominantly intracellular organisms in the upper dermis (periodic acid-Schiff, × 1000); and (d) Gomori methamine silver stain confirming transepidermal elimination of extracellular yeasts into the stratum corneum (Gomori methamine silver, × 1000).

The skin biopsy material was submitted in 10% buffered formalin fixative and was processed for routine histopathological examination. The haematoxylin- and eosin-stained sections revealed lesional skin and subcutaneous tissue harbouring large numbers of small extracellular and intracellular budding yeast cells, the latter contained within macrophages. The small globose and oval fungal yeasts measured approximately 2 μm – 7 μm in diameter, and some showed apparent narrow-based budding. Occasional lymphocytes and neutrophils were also evident amid the background necrotic and karyorrhectic cellular debris (Figure 2a). The overlying epidermis demonstrated striking pseudo-epitheliomatous hyperplasia, with intra-epidermal organisms and transepithelial elimination of the fungi (Figure 2b). These organisms were highlighted on histochemical staining with periodic acid-Schiff (Figure 2c) and Grocott methenamine silver (Figure 2d). Additional sections from the biopsy material were subsequently referred for DNA extraction and pan-fungal polymerase chain reaction (PCR) employing DNA barcoding via the nuclear ribosomal internal transcribed spacer region; this technique is sometimes successful on formalin-fixed, paraffin wax–embedded tissue samples when large numbers of organisms are present.^12^ Unfortunately, the aforementioned investigation proved unsatisfactory as a result of DNA degradation. A presumptive diagnosis of *Emergomyces* sp. infection was nevertheless rendered, based on the light microscopic findings.

Treatment with intravenous amphotericin B was commenced at a dose of 1 mg/kg per day. She received 10 days of treatment with the aforementioned drug but was then sent home on high-dose oral fluconazole (1200 mg/day) after she requested an unauthorised discharge from hospital. Upon the patient’s return to hospital three weeks later, the dose of fluconazole was changed to 200 mg/day as maintenance therapy. Viral load results confirmed that she was failing her first line ART, as evidenced by a VL of 98 970 copies/mL. She was then switched to a second-line ART regimen (azidothymidine, lamivudine, lopinavir and ritonavir). Three months into her antifungal treatment, most of the facial lesions had healed, with residual scars and presence of lesions only around the mouth and nose (Figure 1b). Seven months later, the patient showed clinical improvement, with an increase in her BMI to a normal level (22) and a VL of 82 copies/mL. The fungal infection continued to improve on oral fluconazole therapy (Figure 1c), and at 1-year post-treatment, the facial skin lesions had resolved completely, with minimal scarring (Figure 1d). No significant side effects were reported during the course of treatment.

## Discussion

The dimorphic fungi, *Emmonsia* species, had long been identified as a cause of disease in lower mammals and limited pulmonary disease in immunocompetent individuals.^13^ Emmonsiosis had affected both HIV-infected and HIV-uninfected patients worldwide,^14^ but there was much to be learned about its presence in sub-Saharan Africa.^8^ In recent years, emergomycosis (formerly thermotolerant) caused by the novel thermodependent dimorphic fungus was identified in South Africa as a cause of disseminated disease in individuals living with advanced HIV infection. To date, the only African countries in which cases of *Es. africanus* infection have been documented are South Africa and Lesotho and together with *Es. Pasteurianus*, which was diagnosed in Uganda (in a patient originally from Rwanda), they are the most commonly isolated *Emergomyces* from the African region.^15^

Cutaneous and pulmonary involvements are common in patients infected by *Es. africanus*. In a South African series, 50 of the 52 cases had skin lesions. Other organ systems are also involved, however including the liver, bone marrow, gastro-intestinal tract and lymph node.^3^ As exemplified by the present case, dermatological lesions are usually misdiagnosed as other conditions, such as varicella, papular eruption of HIV, Kaposi sarcoma, pyoderma gangrenosum, drug reactions, scrofuloderma, cutaneous tuberculosis or secondary syphilis.^3,16^ This presumptive case is the second case reported from Lesotho; the first was diagnosed in South Africa and formed part of the series alluded to.

Like almost all of the patients reported from South Africa, who were severely immunocompromised and who had skin lesions, this patient had extensive mucocutaneous disease and virological failure at presentation, with a high VL. Although the diagnosis may be suggested on skin biopsy, reported to be an adequate diagnostic tool, fungal culture is the gold standard for accurate confirmation of the diagnosis.^9^ While not a requirement for all fungal infections, molecular confirmation is a necessary adjunct in most cases of *Emergomyces* infection in order to avoid misdiagnosis in view of its clinical and morphological similarities to histoplasmosis.^14^ Unfortunately, fungal culture and molecular confirmation were not possible in this patient. Histopathological examination of the skin biopsy and attempted retrospective pan-fungal PCR confirmation of the aetiological agent were carried out at a private laboratory in South Africa. The authors acknowledge that histopathology alone is not reliable for a definitive diagnosis of emergomycosis. Other mycoses endemic to sub-Saharan Africa (e.g. histoplasmosis, blastomycosis) share similar histological features, occur with increased frequency among HIV-infected people and frequently exhibit cross-reactivity with antigen tests employed for the diagnosis of emergomycosis. It is also noteworthy that cases because of *Es. pasteurianus* have also been reported from Africa.

Although the patient had mild hypokalaemia upon initiation of amphotericin treatment, the serum potassium concentration returned to normal with correction and maintenance therapy, and no renal impairment was observed. A chest radiograph showed no signs of pulmonary involvement. A bone marrow biopsy was not performed.

Currently, there are no separate management guidelines for emergomycosis. Consequently, general guidelines for invasive mycoses are followed, with recommendation of amphotericin B and a triazole, with good potency.^17,18^ Fluconazole is more available and cheaper but is generally not recommended for treatment because of limited susceptibility.^19,20^ This patient received 10 days of amphotericin B and was sent home mistakenly on a large dose of oral fluconazole (1200 mg/day), following a request for unauthorised discharge. The plan was to complete the two weeks of amphotericin B (4–5 days) with large dose of fluconazole and then to continue with a maintenance dose of 200 mg/day for one year. Itraconazole was unavailable at the time in Lesotho.

## Conclusion

The full geographic extent of emergomycosis in Africa and other parts of the world has yet to be clearly defined. There are several challenges to the diagnosis of the disease, particularly in resource-constrained settings where confirmatory molecular diagnostic tools are not readily available.
